# Impact of spring rape varieties on protein extraction from press cake, emulsifying properties and antinutrient content

**DOI:** 10.1002/jsfa.70547

**Published:** 2026-02-26

**Authors:** Cecilia Ahlström, Evangelia Papagianni, Eu Sheng Wang, Inger‐Cecilia Mayer Labba, Ann‐Sofie Sanberg, Emil Andersson, Johan Thuvander, Karolina Östbring

**Affiliations:** ^1^ Department of Processes and Life Science Engineering Faculty of Engineering, Lund University Lund Sweden; ^2^ Department of Plant Protection Biology Swedish University of Agricultural Sciences Alnarp Sweden; ^3^ Department of Life Sciences Food and Nutrition Science, Chalmers University of Technology Gothenburg Sweden

**Keywords:** rapeseed, protein extraction, emulsions, glucosinolates, phytic acid

## Abstract

**BACKGROUND:**

Rapeseed is the second most cultivated oilseed globally after soybean and a protein‐rich press cake is generated after liberation of the oil. However, its use in food applications is limited by high levels of anti‐nutrients such as glucosinolates and phytic acid. Although differences between botanical varieties are known, controlled comparisons of modern spring varieties for protein functionality and antinutrient reduction are scarce.

**RESULTS:**

Protein was extracted from five spring rape varieties by the pH shift method, yielding 29–37% recovery. The variety Edda exhibited the best emulsifying capacity, stabilizing emulsion droplets of 44 μm (*d*_43_). Glucosinolate concentrations in the protein‐rich precipitates were reduced by 89–94% compared to the press cakes, with Sigrid showing the lowest concentration (0.45 g kg^−1^). Phytic acid content decreased by 53–81% during the extraction, with Fergus having the lowest concentration (7.6 μmol g^−1^).

**CONCLUSION:**

The study demonstrates that emulsifying properties and anti‐nutritional content differ significantly between spring rape varieties. Selecting suitable cultivars, such as Edda for emulsification or Fergus and Sigrid for reduced anti‐nutrients, can improve rapeseed protein functionality for food applications. Future studies should include multiple harvest years to strengthen these findings. © 2026 The Author(s). *Journal of the Science of Food and Agriculture* published by John Wiley & Sons Ltd on behalf of Society of Chemical Industry.

## INTRODUCTION

Rapeseed (*Brassica napus* L., *Brassica rapa* and *Brassica juncea* of rapeseed quality) is the second most commonly cultivated oilseed after soybean^1^ and, in 2022, 80 million tons of rapeseed were harvested globally.^2^ When rapeseed oil is produced, a protein‐rich by‐product is generated. This by‐product is referred to as rapeseed meal if the seeds are hot‐pressed, and rapeseed press cake if cold‐pressed.

Rapeseed protein has a well‐balanced amino acid composition^3^ and could therefore be used as a good source of protein in food applications. The functional properties such as emulsifying properties of rapeseed protein isolates and concentrates have been investigated in multiple studies because these properties govern the product types in which rapeseed protein can be incorporated. Generally, rapeseed proteins have been reported to show great emulsifying properties, which were superior to other protein sources such as soy or flaxseed.^4^ Values reported have been shown to depend on the raw material used for oil extraction and the protein isolation method used. Emulsifying properties such as emulsion capacity and emulsion stability have been reported to be dependent on extraction pH (which govern protein solubility), as well as emulsion pH and ionic strength in the emulsion formulation.^5^, ^6^, ^7^, ^8^ In most studies, cruciferin, one of the storage proteins in rapeseed, has been shown to stabilize emulsions with small droplet size and high stability.^5^, ^9^, ^10^

Rapeseed meal and press cake are commonly used as animal feed or agricultural fertilizer,^8^ whereas the protein in the cold‐pressed meal can be used for the development of high value‐added products.^11^, ^12^ However, the nutritional quality of rapeseed products depends largely on anti‐nutritional factors such as glucosinolates, phytic acid, tannins and sinapinic acid, which currently restricts their application.^13^, ^14^, ^15^ Glucosinolates (GSLs) are secondary plant metabolites derived from different amino acids, such as tryptophan, phenylalanine and methionine, and they also contain sulfate and thioglucose moieties.^16^ Three groups of GSLs are found in oilseed rape: indole (tryptophan‐derived), aromatic (phenylalanine‐derived) and aliphatic.^17^ Glucosinolates and their degradation products play an important role in pest and disease defense reactions on the plant level. On the other hand, high amounts of GSLs in the rapeseed press cake lead to a bitter taste and astringency^18^ and, even more importantly, GSLs can be hydrolyzed to form toxic compounds that interfere with thyroid function resulting in goitrogenic hypertrophy.^19^ Another anti‐nutrient of concern in rapeseed press cake is phytic acid, or phytate as called when present as the deprotonaded anion or in salt form with metal cations such as calcium, magnesium, potassium, iron or zinc. Phytic acid is a naturally occurring compound that stores phosphorous and myoinositol in plants.^20^ It is also considered to store other cations and energy and to protect the plants against oxidative damage during storage and from molds by binding the zinc required for its metabolism.^21^ Phytic acid can form insoluble complexes with many metal cations (divalent and trivalent) at physiological conditions in the gut where absorption takes place. Their presence can have a detrimental impact on mineral absorption if present in the human diet.^22^, ^23^, ^24^ Furthermore, phytic acid can form protein–phytate complexes in a wide range of pHs, which obstruct the enzymatic degradation of protein in the gastrointestinal tract leading to reduced protein absorption.^25^, ^26^, ^27^

Botanical varieties of rapeseed have been reported to vary in concentrations of anti‐nutritional factors, as well as protein recovery yield and functional properties, of which the latter is a result of differences in seed protein composition.^28^, ^29^, ^30^ Spring rape is well‐suited for regions with colder climate, such as the Nordic countries. In the northern parts, winter rape is difficult to cultivate because of the long and cold winters, making spring rape a more viable alternative. Although differences between botanical varieties are known, controlled comparisons of modern spring varieties for protein functionality and antinutrient reduction are scarce. The present study aimed to investigate the protein recovery efficacy from five botanical varieties of spring rapeseed, as well as monitor emulsifying properties and content of glucosinolates and phytic acid in the protein‐rich precipitate.

## MATERIALS AND METHODS

### Materials and chemicals

Five varieties of spring rapeseed were a kind gift from Lantmännen Jordbruk AB (Stockholm, Sweden) where three varieties were hybrid sorts (Edda, Greta and Majong) and two were line sorts (Fergus and Sigrid). The seeds were produced in the same year (2020) at the same location (Lantmännen Jordbruk AB, Svalöv, Sweden) and sourced from the same replicates.

Cold‐pressed rapeseed press cake (references) was a gift from Gunnarshögs Jordbruk AB (Hammenhög, Sweden). The reference was a mixed blend of different winter rape varieties including Alegria, Epure and a few that are unknown. All spring rapeseed varieties and the reference mix were pressed separately in a benchtop oil press at Gunnarshögs Jordbruk AB. The reference mix was also pressed in an industrial press at the same company. Oil pressing was conducted without the use of solvents and the oil temperature was not exceeding 35 °C during pressing. The benchtop oil press was equipped with a heating collar to increase the liberation of oil, with a press cake exit temperature of around 65–70 °C, whereas the temperature of the press cake of the industrial press was 50–55 °C. The press cakes were stored in the freezer (−18 °C) until the start of the protein recovery process.

Citric acid (C_6_H_8_O_7_, CAS 77‐92‐9), sodium chloride (NaCl, CAS 7647‐14‐5), sodium dihydrogen phosphate monohydrate (H_2_NaPO_4_·H_2_O, CAS 7558‐80‐7), disodium hydrogen phosphate dodecahydrate (Na_2_HPO_4_·12H_2_O, CAS 10039–32‐4) and sodium hydroxide (NaOH, CAS 1310‐73‐2) were purchased from Merck (Darmstadt, Germany). Miglyol 812 was purchased from Sasol AG (Hamburg, Germany). All other chemicals were of analytical grade.

### Isolation of protein from rapeseed press cake

**Figure 1:**
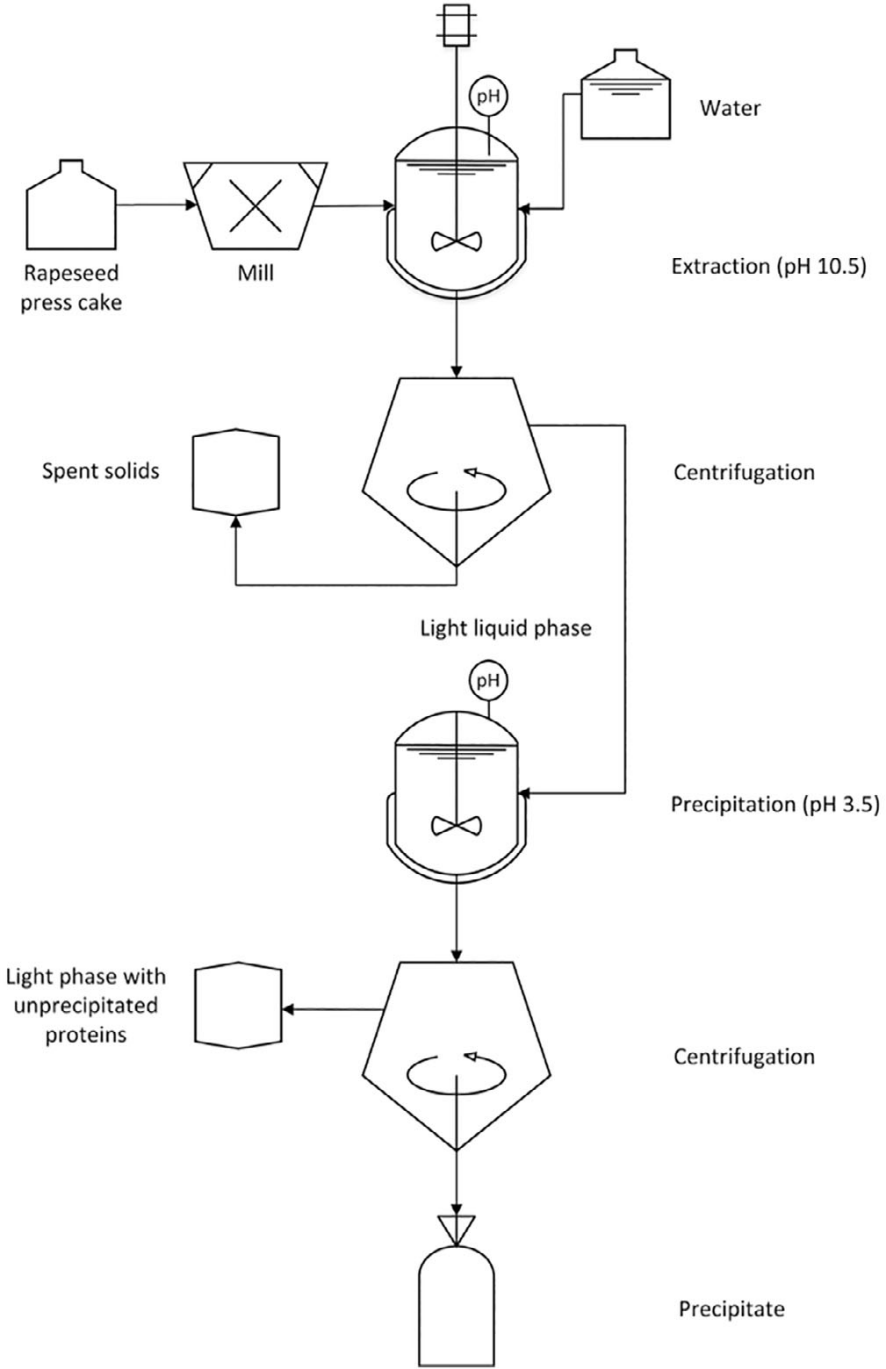
Flowchart illustrating the rapeseed protein isolation process.

Protein extraction was performed according to Ahlström *et al*.^31^ illustrated in Fig. 1. Rapeseed press cake (300 g) was ground in a mixer (R302 VV.; Robot Coupe, Paris, France) for 3 min. The press cake was dispersed in tap water (1:10 w/w), pH was adjusted to 10.5 with 2 m NaOH and the pH was held constant during 1 h of continuous stirring (Eurostar digital; IKA Labortechnik, Staufen, Germany) at 750 rpm. A leaching pH of 10.5 was selected as a compromise to balance protein recovery with the risk of corrosion of stainless steel equipment, and to minimize detrimental effects on protein functionality. After incubation, the dispersion was separated by centrifugation (Allegra® X‐15R Centrifuge; Beckman Coulter, Brea, CA, USA) at 5000 × *g* for 10 min at 20 °C. The supernatant was collected, and the pH was adjusted to pH 3.5 with citric acid followed by centrifugation as described above and the precipitates were collected. After extraction, the subsequent precipitates were freeze‐dried (freeze dryer CD 12;m Hetosicc, Birkerod, Denmark). The material was distributed into aluminum trays to form a 10‐mm layer at maximum and was thereafter frozen at −18 °C for 24 h before freeze‐drying. The plate temperature was 20 °C, the condenser was −50 °C and the vacuum pressure of the dryer was 0.02 mbar. The residence time for the samples in the freeze‐dryer was 7 days. Extractions were performed in duplicate for each spring rapeseed variety, as well as for the two references.

### Proximate analysis

Moisture content was determined by drying the rapeseed precipitate samples at 105 °C for 16 h, according to AOAC 934.01.^32^ The protein concentration of the dehydrated rapeseed precipitates was determined according to the Dumas combustion method AOAC 990.03.^33^ Nitrogen content was determined by the elemental analyzer Flash EA 1112 (Thermo Electron Co., Waltham, MA, USA) blanked with air and with aspartic acid as reference. Approximately 25 mg of material was placed in a tin cylinder (diameter of 30 mm) for analysis and a conversion factor of 6.25 was used to calculate total protein content. Total fat content was determined by solvent extraction in a semi‐automatic Soxtec apparatus (Tecator AB, Höganäs, Sweden), using petroleum benzine as solvent according to AOAC 920.39.^34^ For ash content determination, samples were transferred to porcelain crucibles and incinerated in a furnace at 700 °C for 16 h, a modification of AOAC 923.03.^35^ Carbohydrate content was calculated by difference. All analyses were performed in triplicate.

### Analysis of functional properties

#### Emulsifying properties

Oil‐in‐water emulsions (33%) were prepared in glass test tubes with a 2‐mL continuous phase (0.005 m phosphate buffer, 0.2 m NaCl, pH 7), 1‐mL dispersed phase (Miglyol 812; Sassol AG, Hamburg, Germany) and freeze‐dried rapeseed precipitate equivalent to 8 mg of protein. Two emulsions were prepared from each process replicate. This protein concentration was chosen based on a preliminary study where different protein concentrations were evaluated and concentrations above 8 mg rapeseed protein mL^−1^ oil did not result in any further reduction in droplet size (data not shown). Freeze‐dried precipitete was added to the phosphate buffer and was allowed to rehydrate for 10 min followed by a pre‐homogenization at 20 000 rpm for 60 s. Miglyol was added and the emulsions were homogenized (D‐79282; Ystral, Ballrechten‐Dottingen, Germany) at 20 000 rpm for 60 s. The emulsions were incubated for 1 h at 4 °C before size measurements. The particle size distribution of the emulsions was analyzed with a laser diffraction particle analyzer (Mastersizer 2000, version 5.60; Malvern Pananlytical, Malvern, UK). The pump velocity was 2000 rpm with 100 mL of MilliQ® water (Merck Millipore Burlington, MA, USA) in the sampling chamber. Each emulsion replicate was measured three times and the average was reported. The refractive index (RI) was 1.45 for the miglyol oil and 1.33 for the water. The obscuration was between 10% and 20%. The volume‐weighted mean diameter (*d*_43_) and particle distribution (volume %) were calculated.

#### Water/oil holding capacities

The water/oil holding capacities of extracted rapeseed precipitates were analyzed as described by Stone *et al*.^36^ Briefly, 5.0 g of water/oil was added to samples (0.5 g) in a 50‐mL graduated centrifuge tube and vortexed for 10 s every 5 min for 30 min. The mixtures where then centrifuged at 1000 × *g* for 10 min. Supernatants were carefully removed, and the precipitates were collected and weighed and water/oil holding capacities were calculated. Analysis was conducted in triplicate.

### Analysis of antinutritional factors

Extraction and quantification by liquid chromatographymass spectrometry (LC–MS) of GSLs was performed according to the method described by Doheny‐Adams *et al*. (2017)^37^ with modifications. Rapeseed press cake and protein‐rich precipitates were kept frozen at −18 °C and freeze‐dried for 48 h. In total, 2 g of the freeze‐dried material was homogenized in a Retsch tissue lyser (MM 200 Model Mixer Mill; Retsch GmbH, Haan‐Gruiten, Germany) at 30 Hz for 1 min and three 50‐mg triplicates were prepared from the milled material in 2‐mL tubes. Any residual lipids in the milled sample were removed by the addition of 1 mL of heptane, followed by a 30‐min incubation in a thermomixer at 1000 rpm. The tubes were then centrifuged at 14 000 rpm and the supernatant was discarded. The defatting procedure was repeated once more with 1 mL of fresh heptane. Residual heptane was removed by drying the pellet in a vacuum desiccator (Eppendorf, Hamburg, Germany) for 10 min at 45 °C. Total GSLs was extracted via methanol extraction according to Doheny‐Adams *et al*. (2017).^37^ In summary, 1 mL of 70% methanol was added to each sample and the pellet was re‐suspended by vortexing for 1 min. Samples were then incubated at 75 °C for 30 min. The supernatant now containing GSLs was recovered following centrifugation at 14 000 rpm for 5 min and transferred to a 2‐mL screwcap tube. Another 1 mL of fresh 70% methanol was added to the residual pellet and the extraction procedure was repeated once more. The supernatant recovered from the second extraction was pooled together with the earlier collect, and GSL methanol extracts were stored at −20 °C until analysis.

Prior to analysis via LC–MS, a 100‐μL aliquot of the methanol extract was transferred to a new 2 mL tube and evaporated in a vacuum desiccator (Eppendorf) for 15 min at 30 °C or until dry. The dried samples were reconstituted with 1 mL of deionized Millipore‐quality water and centrifuged at 14 000 rpm to pellet any residual particulate matter. A minimum of 600 μL of the clarified supernatant was transferred to a glass HPLC vial for analysis. The identification and quantification of individual GSLs was achieved via HPLC using an Agilent 1260‐MS/Agilent 6120‐ESI system (Agilent, Santa Clara, CA, USA) in negative mode. Separation of GSLs was achieved through a Luna 3 μm C18(2) column (Phenomenex, Torrance, CA, USA) with 0.1% formic acid (A) in MilliQ water and 100% acetonitrile (B) as eluants with a constant flow rate of 0.35 mL min^−1^. The mobile phase program was 100% A for 4 min, followed by a 10‐min linear gradient to 70% A and 30% B for an additional 1 min, followed by a 1‐min linear gradient back to 100% A for an additional 3 min. Individual GSLs were identified and quantified by comparing their retention times, peak areas and molecular mass in relation to standard curves generated from certified standards of progoitrin, glucoiberin, glucoraphanin and neoglucobrassicin (ChemFaces, Wuhan, China). Analysis was performed in triplicate.

Freeze‐dried precipitate of rapeseed press cake were analyzed for phytic acid (inositol hexaphosphate, InsP6) by HPLC.^38^, ^39^ The samples (0.5 g) were extracted with 10 mL of 0.5 mol L^−1^ HCl for 3 h using a laboratory shaker (Heidolph Reax 2; Heidolph Instruments GmbH, Schwabach, Germany). Next, 1 mL was removed, centrifuged, filtered using a syringe filter with 0.45 μm pore size (PES‐membrane, Fischerbrand; Thermo Fisher Scientific, Waltham, MA, USA) to remove oil and the supernatant was transferred to an HPLC vial. The chromatography setup consisted of an HPLC pump (model PU‐4080i; Jasco Inc., Easton, MD, USA) for the eluent and an RHPLC pump (model PU‐4180; Jasco Inc.) equipped with a PA‐100 guard column and a CarboPac PA‐100 column (Thermo Fisher Scientific). InsP6 was eluted with an isocratic eluent of 80% HCl (1 mol L^−1^) and 20% H_2_O at 0.8 mL min^−1^, subjected to a post‐column reaction with ferrous nitrate, and detected at 290 nm in a UV‐visible HPLC detector (UV‐4075; Jasco Inc.). Each sample had a run time of 7 min, and the InsP6 concentration was calculated using external standards covering the concentration range of 0.1–0.6 μmol mL^−1^. Analyses were performed in duplicate.

### Statistical analysis

Statistical analyses were performed using SPSS, version 26 (IBM Corp., Armonk, NY, USA). One‐way analysis of variance with Tukey's post‐hoc test was used to investigate significant differences. *P* < 0.05 was considered statistically significant. All results are expressed as the mean ± SD.

## RESULTS AND DISCUSSION

### Composition and protein recovery yield

Press cake from the five spring rapeseed varieties had similar dry solids content of around 920 g kg^−1^ and no statistical differences could be found. The protein concentration ranged from 321 to 356 g kg^−1^, with Sigrid exhibiting significantly higher concentration than the others (Table 1). The fat content was in general similar for the rapeseed varieties but Fergus contained significantly more fat compared to Edda, Majong and Sigrid. The carbohydrate content varied between 383 and 419 g kg^−1^ and the ash content 45–47 g kg^−1^. Greta had lower ash content than Edda and Fergus, whereas Majong and Sigrid did not differ from the other individual varieties. In comparison with the winter rape references, the fat content in RefIP was lower (112 g kg^−1^) which was the result of a more efficient oil pressing (see Supporting information, Table S1).

**Table 1:** Proximate analysis on a dry basis of rapeseed press cakes from different spring rapeseed varieties.

Rapeseed variety	Dry solids (g kg^−1^)	Protein (g kg^−1^)	Fat (g kg^−1^)	Carbohydrates (g kg^−1^)	Ash (g kg^−1^)
Rapeseed press cake					
Edda	924 *±* 4 a	337 *±* 2 bc	212 ± 3 a	404 ± 4 bc	47 ± 0 b
Fergus	924 *±* 2 a	329 *±* 3 ab	226 ± 1 b	398 ± 3 ab	47 ± 0 b
Greta	918 *±* 5 a	321 *±* 5 a	216 ± 6 ab	419 ± 10 c	45 ± 0 a
Majong	918 *±* 0 a	343 *±* 2 c	214 ± 2 a	397 ± 2 ab	46 ± 0 ab
Sigrid	920 *±* 1 a	356 *±* 7 d	215 ± 5 a	383 ± 3 a	46 ± 1 ab

*Note*: Carbohydrates are calculated by difference and data are given as the mean ± SD. Different lowercase letters in columns indicate a significant difference (*P* < 0.05).

After protein extraction, the extraction and precipitation coefficients were calculated indicating how large proportion of the protein in the press cake that was extracted to the aqueous phase and thereafter precipitated into the sediment. The extraction coefficient was found to vary between 57% and 61% and the precipitation coefficient between 51% and 61%, but no statistical difference was found (Table 2). The protein recovery yield ranged from 29% to 37% and there was no significant difference between the individual varieties. The protein recovery yield for the winter rape reference pressed in an industrial press was significantly higher (43%) than the spring rape varieties, whereas no difference could be found for the winter rape reference pressed in the benchtop press (see Supporting information, Table S2). Because the industrial press exposes the press cake to lower temperatures than the benchtop press, the results indicate that maintaining as low a temperature during oil pressing is important for achieving a high yield. The protein recovery yield for the spring rape varieties in the present study was lower than reported by Fetzer *et al*.^40^ (40.6%) and in the same range as a study conducted on the isolation of protein from different varieties of winter rapeseed (32–41%).^29^ In studies where higher alkali pH (pH 12) was used in the extraction phase of the process, protein recovery yields of up to 56% were reached.^28^ No difference in protein recovery yield between the botanical cultivars of spring rape could be found in the present study, and it should be noted that the present study was performed with rapeseed varieties from one single year. Further studies including rapeseed varieties from several years should be performed to present more robust data.

**Table 2:** Extraction data and protein recovery yield from different spring rapeseed varieties.

Rapeseed variety	Extraction coefficient (%)	Precipitation coefficient (%)	Protein recovery yield (%)
Edda	61.3 ± 3.5 b	54.6 ± 8.3 a	33.9 ± 0.2 a
Fergus	59.1 ± 3.3 ab	50.7 ± 7.8 a	29.1 ± 0.3 a
Greta	57.0 ± 6.3 ab	59.4 ± 9.1 a	37.0 ± 5.0 ab
Majong	57.3 ± 4.7 ab	55.6 ± 8.3 a	33.2 ± 2.0 a
Sigrid	59.0 ± 5.1 ab	61.0 ± 12 a	35.2 ± 1.8 ab

*Note*: Data are given as the mean ± SD. Different lowercase letters in columns indicate a significant difference (*P* < 0.05).

**Figure 2:**
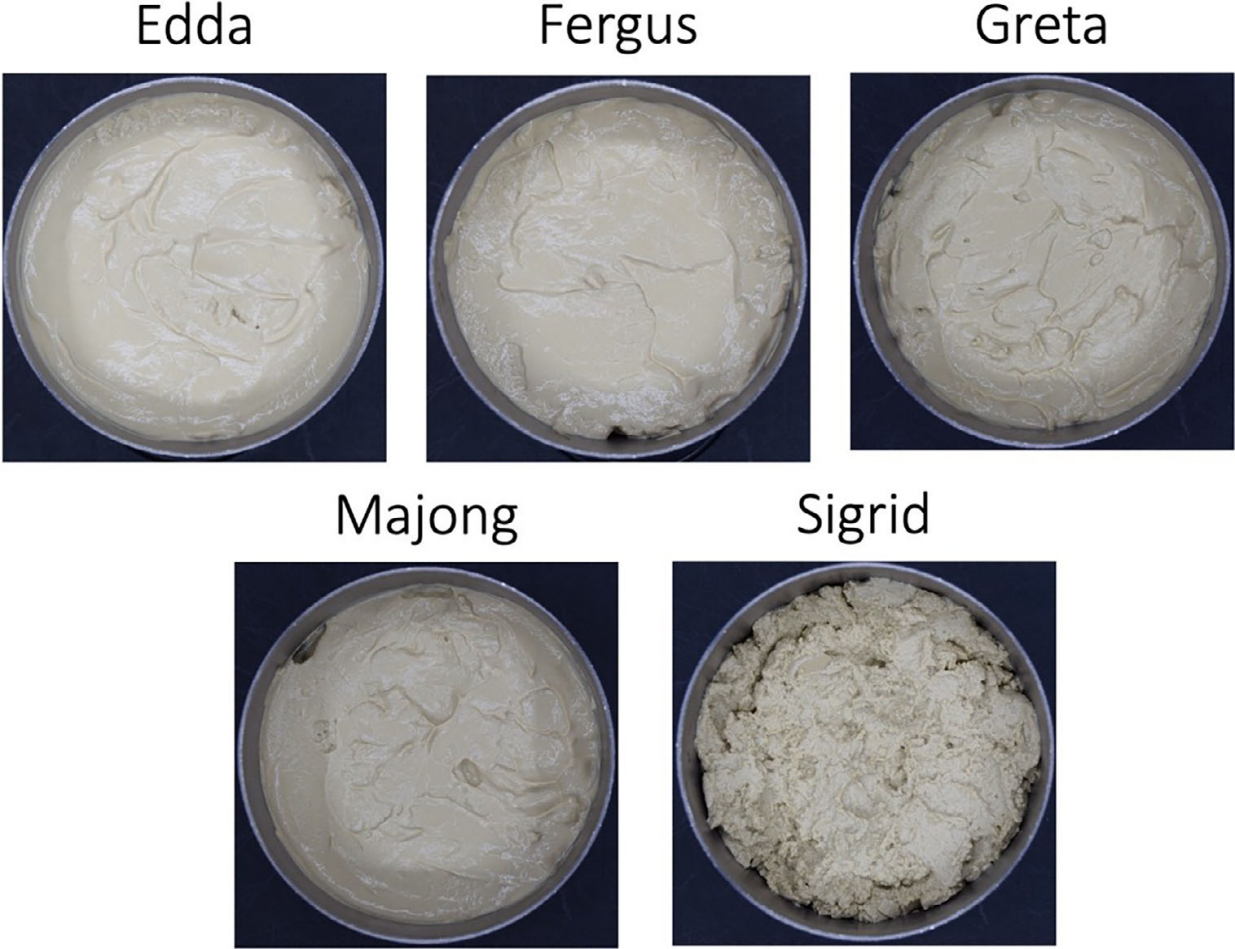
Sediments from rapeseed press cake from different botanical varieties of spring rapeseed.

The resulting precipitates from the different rapeseed press cakes were similar in proximate composition (Table 3). Sigrid had the highest content of dry solids (267 g kg^−1^) and Edda had the lowest content (226 g kg^−1^), although no statistical difference was found between the cultivars. In Fig. 2 (see also Supporting information, Fig. S1), it can be seen that Sigrid was grittier and drier compared to the other samples, which can be linked to the low moisture content for this variety. Protein concentration was ranging between 499 g kg^−1^ for Fergus and 549 g kg^−1^ for Sigrid, without a statistically significant difference. The protein concentration in RefIP (see Supporting information, Table S3) was significantly higher (575 g kg^−1^), emphasizing the importance of controlled temperature during oil pressing to limit heat‐induced protein denaturation. Protein concentration is related to alkali pH in the extraction phase, where a higher pH results in higher protein concentration after protein precipitation. Several studies have investigated the protein concentration in precipitates after extraction at pH 12 followed by precipitation and the protein concentrations range between 760 and 809 g kg^−1^.^28^, ^41^, ^42^ Fetzer *et al*.^40^ used a salt‐based protein recovery method combined with ultrafiltration to concentrate the proteins and reported protein concentrations of 780–840 g kg^−1^ depending on pH in the leaching phase. The precipitates had a fat concentration varying between 309 and 367 g kg^−1^ with Fergus exhibiting the significantly highest fat concentration. Fergus also had the highest fat content in the corresponding press cake. Carbohydrate and ash content in the sediments did not differ between the varieties.

**Table 3:** Proximate analysis on a dry basis of sediments after the protein recovery process.

Rapeseed variety	Dry solids (g kg^−1^)	Protein (g kg^−1^)	Fat (g kg^−1^)	Carbohydrates (g kg^−1^)	Ash (g kg^−1^)
Edda	226 *±* 17 ab	521 *±* 14 ab	335 ± 8 abc	121 ± 7 a	23 ± 2 a
Fergus	236 *±* 34 ab	499 *±* 20 ab	367 ± 4 c	111 ± 23 a	23 ± 4 a
Greta	232 *±* 21 ab	532 *±* 5 bc	322 ± 18 ab	123 ± 23 a	22 ± 2 a
Majong	245 *±* 32 ab	537 *±* 2 bc	331 ± 4 abc	109 ± 21 a	23 ± 1 a
Sigrid	267 *±* 49 b	549 *±* 2 bc	309 ± 36 a	122 ± 54 a	20 ± 2 a

*Note*: Carbohydrates are calculated by difference and data are given as the mean ± SD. Different lowercase letters in columns indicate a significant difference (*P* < 0.05).

### Functional properties

**Figure 3:**
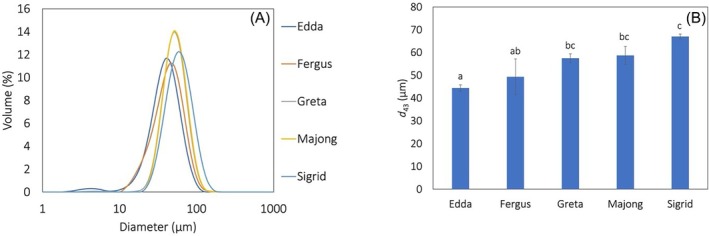
(A) Size distributions and (B) emulsion droplet size (*d*_43_) in emulsions stabilized by rapeseed protein extracted from press cake from different spring varieties. Emulsions were 33% oil‐in‐water emulsions produced by high shear homogenization. Emulsifier concentrations in the emulsions were 8 mg protein mL^−1^ oil and data are an average from four measurements for each formulation with SD. Different letters indicate statistical difference (*P* < 0.05).

The protein recovered from the individual spring rapeseed varieties stabilized emulsions with bimodal droplet size with a dominating peak around 40–65 μm (Fig. 3(A)). Most emulsions displayed a smaller peak around 5 μm representing protein aggregates in the continuous phase, which was also observed by Chang *et al*.^43^, and Pirestani *et al*.^44^ Edda had the highest emulsifying capacity and stabilized emulsions with a smaller volume‐weighted mean droplet diameter (40–42 μm) than protein extracted from the other spring rapeseed varieties (44–67 μm), except Fergus (Fig. 3(B)). In our previous study using the same emulsion model, protein from individual cultivars of winter rape stabilized emulsion droplets with *d*_43_ of 42–51 μm, which is slightly smaller than the present study.^29^ Tan *et al*.^45^ reported the *d*_43_ to be around 60 μm for rapeseed protein precipitated at pH 4.0. In the study by Tan *et al*.^45^, pH 12 was used in the extraction phase compared to the milder extraction conditions in the present study, where pH 10.5 was used. Extreme alkali treatment has been reported to affect the emulsifying properties of rapeseed protein negatively due to denaturation of protein.^46^

Protein extracted from the two winter rape references exhibited smaller emulsion droplets (40–42 μm) (see Supporting information, Fig. S2) than the spring rape varieties. It was found that winter rapeseed proteins generally stabilize smaller emulsion droplets than those from spring varieties. This may be related to biochemical adaptations during cold acclimation, such as the accumulation of dehydrins which are highly hydrophilic, amphipathic proteins that protect cell membranes under freezing and dehydration stress.^47^, ^48^ Differences in protein composition and structure between spring and winter rapeseed likely explain the higher interfacial affinity and improved emulsifying ability observed in winter rapeseed proteins. This hypothesis should be further investigated in future studies.

The water and oil holding capacities (WHC and OHC) of the spring rape varieties were found to be in the range of 308–329% and 242–277%, respectively, but no statistical difference could be found between the sediments (Table 4; see also Supporting information, Table S4). This was expected because the proximate composition was very similar. The only difference found was that protein extracted from winter rape pressed in a benchtop press had significantly higher WHC compared to the other (428%) (see Supporting information, Table S4). This was unexpected because WHC is often positively correlated to concentration of carbohydrates (especially fibers) and protein and negatively correlated to fat.^49^ In the present study, the RefBP had lower concentration of carbohydrate (although not significant), lower concentration of protein and higher concentration of fat compared to RefIP. Fiber was not specifically analyzed, and no conclusion can therefore be drawn regarding how carbohydrate composition affected the WHC, such that this should be further investigated. The WHC observed in the present study was lower than that reported by Li *et al*. (366%), likely a result of the lower protein concentration in their samples (31%), whereas the OHC was in the same range (263%).^49^

**Table 4:** Water holding capacity (WHC) and oil holding capacity (OHC) of sediments after the protein recovery process. Data are given as the mean ± SD.

Rapeseed variety	WHC (%)	OHC (%)
Edda	329 ± 16 a	266 ± 9.1 a
Fergus	317 ± 11 a	265 ± 18 a
Greta	308 ± 3.4 a	242 ± 9.2 a
Majong	315 ± 8.4 a	248 ± 3.6 a
Sigrid	304 ± 7.2 a	277 ± 30 a

*Note*: Different lowercase letters in columns indicate a significant difference (*P* < 0.05).

### Glucosinolates

There were eight individual GSLs as well as an unknown peak detected across the press cake samples and the concentration of each GSL varied between rapeseed variety (Table 5; see also Supporting information, Table S5). Four aliphatic GSLs (progoitrin, glucoraphanin, gluconapoleiferin and gluconapin), three indole GSLs (4‐hydroxyglucobrassicin, glucobrassicin and neoglucobrassicin) and one aromatic GSL (gluconasturtiin) were found. The unidentified compounds corresponding to molecular weights of 389, 448 and 478 g/mol could not be resolved and were all in the same peak. They shared similar relative molecular masses because some of the other identified GSLs but did not have the same retention time as the controls, nor the identified GSL peak. Therefore, we conclude that they are probably unidentified GSL complexes. The GSLs present in the highest amount in the press cakes were progoitrin, 4‐hydroxyglucobrassicin and glukonapin, which agree with previous studies.^50^, ^51^ These three GSLs is common to be dominating in European cultivated rapeseed.

**Table 5:** Concentration of glucosinolates in press cake and sediment after protein recovery from five spring rapeseed varieties.

Glucosinolate type	Edda	Fergus	Greta	Majong	Sigrid
Press cake					
4‐Hydroxyglucobrassicin	2.29 ± 0.1 bc	2.33 ± 0.1 c	2.17 ± 0.1 bc	2.12 ± 0.0 b	2.25 ± 0.1 bc
Glucoarmoracialapicin	–	–	–	–	–
Glucobrassicin	0.06 ± 0.0 ab	0.08 ± 0.0 c	0.07 ± 0.0 b	0.07 ± 0.0 b	0.06 ± 0.0 a
Glucoiberin	–	–	–	–	–
Gluconapin	0.86 ± 0.0 c	0.57 ± 0.0 a	0.54 ± 0.0 a	0.72 ± 0.0 b	0.59 ± 0.0 a
Gluconapoleiferin	–	–	–	0.12 ± 0.0 a	–
Gluconasturtiin	0.19 ± 0.0 e	0.12 ± 0.0 c	0.09 ± 0.0 b	0.11 ± 0.0 c	0.08 ± 0.0 a
Glucoraphanin	0.09 ± 0.0 b	0.02 ± 0.0 a	0.02 ± 0.0 a	0.09 ± 0.0 b	–
Neoglucobrassicin	0.22 ± 0.0 c	0.19 ± 0.0 b	0.44 ± 0.0 d	0.54 ± 0.0 e	0.10 ± 0.0 a
Progoitrin	1.67 ± 0.1 c	1.17 ± 0.0 b	0.94 ± 0.0 a	1.60 ± 0.1 c	1.20 ± 0.0 b
GSLs Mw 389, 448, 478	0.64 ± 0.0 abc	0.62 ± 0.0 ab	0.67 ± 0.0 bcd	0.68 ± 0.0 bcd	0.60 ± 0.0 a
Total GSLs	6.02 ± 0.7 b	5.10 ± 0.7 a	4.95 ± 0.6 a	6.05 ± 0.7 b	4.87 ± 0.7 a
Sediment					
4‐Hydroxyglucobrassicin	–	–	–	–	–
Glucoarmoracialapicin	0.13 ± 0.0 b	0.13 ± 0.0 b	0.13 ± 0.0 b	0.12 ± 0.0 ab	0.12 ± 0.0 a
Glucobrassicin	–	–	–	–	–
Glucoiberin	–	–	–	–	–
Gluconapin	–	–	–	–	–
Gluconapoleiferin	–	–	–	–	–
Gluconasturtiin	–	–	–	–	–
Glucoraphanin	–	–	–	–	–
Neoglucobrassicin	–	–	–	–	–
Progoitrin	–	–	–	–	–
GSLs Mw 389, 448, 478	0.40 ± 0.0 b	0.39 ± 0.0 b	0.42 ± 0.0 b	0.39 ± 0.0 b	0.33 ± 0.0 a
Total GSLs	0.52 ± 0.1 bc	0.51 ± 0.1 bc	0.55 ± 0.1 c	0.51 ± 0.1 bc	0.45 ± 010 a

*Note*: Results are expressed as the mean g kg^−1^ on a dry basis with SD. Dash (−) indicates concentrations below detection limit. Different lowercase letters in columns indicate a significant difference (*P* < 0.05).

**Figure 4:**
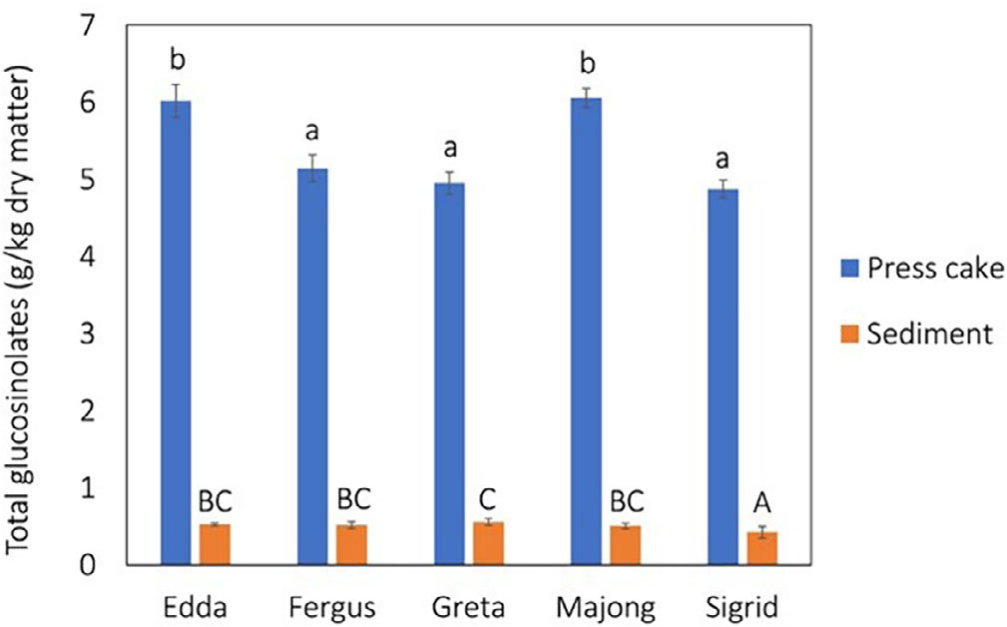
Glucosinolates concentration in press cake and corresponding freeze‐dried sediment after protein recovery. Data are given as the mean ± SD. Lowercase letter indicates statistical difference between press cakes (*P* < 0.05) and uppercase letter indicates statistical difference between sediments (*P* < 0.05).

The concentration of total GSLs differed between rapeseed press cakes from different individual rapeseed varieties (Fig. 4). The lowest concentrations (4.95–5.10 g kg^−1^) were found for the varieties Fergus, Greta and Sigrid, whereas Edda and Majong had higher concentrations (6.02–6.05 g kg^−1^). The highest concentration was found in press cake from industrially pressed winter rape (7.96 g kg^−1^) (Fig. S3). The reason is probably the lower fat content in this press cake. The concentrations of total GSLs in press cakes were slightly higher than the results reported by others where Tan *et al*.^30^ reported concentrations of 2.9 mg GSLs g^−1^ in hot‐pressed rapeseed meal and Tzeng *et al*.^41^ found 3.1 mg GSLs g^−1^ in the industrially hot‐pressed meal. The concentration range in the present study is in line with concentrations found by Tzeng *et al*.^52^ who found the GSL concentration to be 4.8 mg GSLs g^−1^ in laboratory prepared meals.

The variety Sigrid had the significantly lowest concentration of GSLs after protein recovery (0.45 g kg^−1^) compared to the other investigated spring varieties. Greta had low values in the press cake but showed the highest GSLs concentration in the protein‐rich precipitate (0.55 g kg^−1^ GSLs), although not significant. The concentrations of GSLs in the sediments corresponds to a reduction of 89–94% of the initial concentrations in the press cakes (Fig. 4; see also Supporting information, Fig. S3). Only glucoarmoracialapicin and GSLs Mw 389, 448, 478 g/mol were present in the protein‐rich precipitates, all other of the investigated glucosinolate types were completely removed. Water treatments such as soaking and water extraction are among the most effective methods for reducing the GSL content of rapeseed meals.^53^ The results are higher than previous studies where rapeseed protein isolates have been found to have less than 0.06 mg GSLs g^−1^.^52^ Chmielewska summarized commercial rapeseed protein isolates with GRAS approval and concentrations of GSLs were found to be in the range 0.25–0.39 mg g^−1^.^20^

### Phytic acid

**Figure 5:**
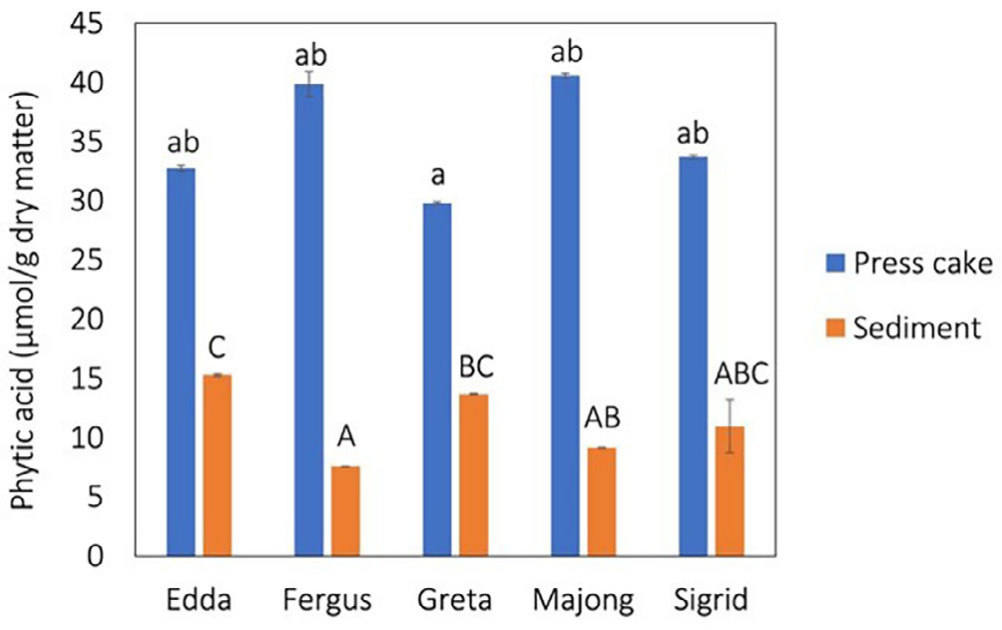
Phytic acid in press cake and corresponding freeze‐dried sediment after protein recovery. Data are given as the mean ± SD. Lowercase letter indicates statistical difference between press cakes (*P* < 0.05) and uppercase letter indicates statistical difference between sediments (*P* < 0.05).

The initial phytic acid concentration in the press cakes ranged between 30 and 45 μmol g^−1^ with Greta showing the significantly lowest concentration (Fig. 5). Common concentrations in rapeseed meal are 30–75 μmol g^−1^.^54^ After protein extraction, the phytic acid concentrations in the precipitates were significantly decreased to 8–24 μmol g^−1^, which corresponds to a 53–81% reduction (Fig. 5). Fergus expressed the lowest concentration of phytic acid of the varieties investigated, although no significant difference could be found between Fergus, Majong and Sigrid. Despite Fergus and Majong having the highest initial concentrations in the press cakes, these two varieties expressed the lowest content of phytic acid in the sediment. Phytic acid concentrations for the winter rape references were higher both in rapeseed press cake and the corresponding sediment (see Supporting information, Fig. S4).

The findings for the individual varieties are in line with Dipak *et al*.^55^ who reported 0.7% phytic acid (corresponding to 10.5 μmol g^−1^) in their precipitates. Rodrigues *et al*.^54^ reported lower concentrations of phytic acid in their study, 0.1% (1.5 μmol g^−1^) and the results were explained by both an unusual low starting concentration in the rapeseed meal (20 μmol g^−1^) compared to 33–45 μmol g^−1^ in the present study, as well as the use of the phytic acid degrading enzyme phytase in the protein extraction procedure.^54^ Zhang *et al*.^8^ found that precipitation pH was affecting the final phytic acid content in the protein‐rich precipitate, where phytic acid increased with reduced pH. They reported the phytic acid concentration to be 5.3 μmol g^−1^ when using precipitation pH of 3.5 and a reduction to 3.4 μmol g^−1^ only by elevating the precipitation pH to 4.0. Another study on hemp protein extraction conducted in our laboratory confirmed the findings of Zhang *et al*.^8^, where phytic acid concentration in the protein‐rich precipitate had a minimum at a higher precipitation pH than the preferred highest protein yield.^56^ There appears to be a conflict between protein recovery yield and phytic acid concentration in terms of precipitation pH, and both cannot be optimized in the same process. Processing strategies to reduce phytic acid on an industrial scale are not fully established, but the use of phytase has shown promising results in laboratory‐scale studies,^54^ although no food‐grade phytase is currently available. Because of the conflict between minimizing the phytic acid and maximizing the protein yield, several measures aside from monitoring the pH must be taken. In the present study, a precipitation pH of 3.5 was used because previous studies in our laboratory have shown this particular pH to give the highest precipitation yield.

Many plant protein concentrates and isolates are used in meat substitutes and Mayer Labba *et al*.^57^ found the estimated bioavailability of minerals to be poor in the majority of the 44 investigated commercial food products on the Swedish market. The reason was the high content of phytic acid, which can chelate iron and form insoluble aggregates, inaccessible for absorption by the human gastrointestinal tract, resulting in poor absorption of iron. The inhibiting effect of phytic acid on iron absorption occurs already at very low phytate levels and has a significant effect at 54 μmol in a meal.^22^ This equals 7.0 g of extracted protein sediment (dry weight) of Fergus, which had the lowest phytic acid content. The inhibiting effect of phytic acid on iron absorption can be partly counteracted by adding enhancing factors, comprising ascorbic acid or the meat factor, to the meal,^23^ but the effectiveness depends on the molar ratio between the enhancers and inhibitors, as well as the form and concentration of the iron. A phytic acid: iron molar ratio ≤ 6 is widely proposed as a cutoff where the inhibiting effect of phytic acid can be counteracted with dietary enhancers. Applying this criterion on a high moisture meat analogue with 60% moisture content, and an iron content of 2.1 mg per 100 g, which is the lower limit where a nutrition claim on iron can be made in the European Union, up to 70% of the dry matter can originate from the Fergus protein sediment, whereas only 25% would be acceptable using the precipitate with the highest phytic acid content. Therefore, it is important to both have control over the concentration of phytic acid in alternative protein concentrates and isolates and reduce the levels to as great extent as possible, especially in fully plant based products. Because heme iron is not affected by phytic acid, hybrid products that include both rapeseed protein extract and animal protein could help maintain iron bioavailability. The present study shows that there are differences in phytic acid depending on rapeseed varieties, with Fergus exhibiting the lowest concentration among the investigated botanical varieties.

## CONCLUSIONS

Protein was extracted from rapeseed press cake of five spring rape varieties and the protein‐rich precipitates were assessed for protein recovery yield, emulsifying properties, as well as concentration of glucosinolates and phytic acid. No significant difference in yield or protein concentration in the precipitates could be detected between the spring rape varieties, but Fergus had higher fat content. Edda showed the highest emulsifying capacity and stabilized smaller emulsion droplets compared to the other spring rape varieties. Glucosinolate concentrations were reduced by 89–94% after protein extraction, with the spring variety Sigrid showing the lowest residual content (0.45 g kg^−1^). Phytic acid was decreased by 53–81% and Fergus had the lowest concentration in the precipitate (7.6 μmol g^−1^). The present study demonstrates that the choice of spring rape cultivar allows for the targeted production of protein isolates: (i) Edda for applications requiring superior emulsification and (ii) Sigrid or Fergus for applications where minimal glucosinolate or phytic acid content is a priority. Future studies including multiple harvest years are recommended to confirm these findings.

## AUTHORS CONTRIBUTIONS

CA, ESW, I‐CML, A‐SS and EA were responsible for formal analysis. CA was responsible for data curation.

EP was responsible for statistical analysis. JT was responsible for reviewing and editing. JT and KÖ were responsible for supervision.

KÖ was responsible for conceptualization. KÖ was responsible for methodology. KÖ was responsible for writing the original draft.

## CONFLICTS OF INTEREST

The authors declare that they have no conflicts of interest.

## Supporting information


**Figure S1.** Sediments from rapeseed press cake. RefBP and RefIP are sediment extracted from press cake from a mixed blend of different rapeseed varieties where BP indicates that a benchtop oil screw press was used in the liberation of oil and IP indicates that an industrial oil screw press was used to generate the starting material.
**Figure S2.** Size distributions and emulsion droplet size (*d*_43_) in emulsions stabilized by rapeseed protein extracted from press cake from different spring varieties. Emulsions were 33% oil‐in‐water emulsions produced by high shear homogenization. Emulsifier concentrations in the emulsions were 8 mg rapeseed protein mL^−1^ oil and data are an average from four measurements for each formulation with SD. RefBP = reference rapeseed blend pressed in a benchtop oil press. RefIP = reference rapeseed blend pressed in an industrial oil press.
**Figure S3.** Glucosinolates concentration in press cake and corresponding freeze‐dried sediment after protein recovery. RefBP = reference rapeseed blend pressed in a benchtop oil press. RefIP = reference rapeseed blend pressed in an industrial oil press. Data are given as the mean ± SD.
**Figure S4.** Phytic acid in press cake and corresponding freeze‐dried sediment after protein recovery. RefBP = reference rapeseed blend pressed in a benchtop oil press. RefIP = reference rapeseed blend pressed in an industrial oil press. Data are given as the mean ± SD.
**Table S1.** Proximate analysis on a dry basis of rapeseed press cakes from mixed blends of winter rape as references. Carbohydrates are calculated by difference and data are given as the mean ± SD. RefBP = reference rapeseed blend pressed in a benchtop oil press. RefIP = reference rapeseed blend pressed in an industrial oil press.
**Table S2.** Extraction data and protein recovery yield from winter rape references. Data are given as the mean ± SD. RefBP = reference rapeseed blend pressed in a benchtop oil press. RefIP = reference rapeseed blend pressed in an industrial oil press.
**Table S3.** Proximate analysis on a dry basis of sediments after the protein recovery process. Carbohydrates are calculated by difference and data are given as the mean ± SD. RefBP = reference rapeseed blend pressed in a benchtop oil press. RefIP = reference rapeseed blend pressed in an industrial oil press.
**Table S4.** Water holding capacity (WHC), oil holding capacity (OHC) and solubility of sediments after the protein recovery process. Data are given as the mean ± SD. RefBP = reference rapeseed blend pressed in a benchtop oil press. RefIP = reference rapeseed blend pressed in an industrial oil press.
**Table S5.** Concentration of glucosinolates in press cake and sediment after protein recovery from a mixed blend of winter rape. Results are expressed as the mean g kg^−1^ on a dry basis with SD. RefBP = reference rapeseed blend pressed in a benchtop oil press. RefIP = reference rapeseed blend pressed in an industrial oil press. Dash (−) indicates concentrations below detection limit.

## Data Availability

The data that support the findings of this study are available from the corresponding author upon reasonable request.
