# Supplementary material for: Impact of spring rape varieties on protein extraction from press cake, emulsifying properties and antinutrient content
Source: J Sci Food Agric. 2026 Feb 26;106(8):4613–22. doi: 10.1002/jsfa.70547 (PMC13157242; doi:10.1002/jsfa.70547)
Supplement: Supplementary file 1 — Figure S1. Sediments from rapeseed press cake. RefBP and RefIP are sediment extracted from press cake from a mixed blend of different rapeseed varieties where BP indicates that a benchtop oil screw press was used in the liberation of oil and IP indicates that an industrial oil screw press was used to generate the starting material. Figure S2. Size distributions and emulsion droplet size (d 43) in emulsions stabilized by rapeseed protein extracted from press cake from different spring varieties. Emulsions were 33% oil‐in‐water emulsions produced by high shear homogenization. Emulsifier concentrations in the emulsions were 8 mg rapeseed protein mL−1 oil and data are an average from four measurements for each formulation with SD. RefBP = reference rapeseed blend pressed in a benchtop oil press. RefIP = reference rapeseed blend pressed in an industrial oil press. Figure S3. Glucosinolates concentration in press cake and corresponding freeze‐dried sediment after protein recovery. RefBP = reference rapeseed blend pressed in a benchtop oil press. RefIP = reference rapeseed blend pressed in an industrial oil press. Data are given as the mean ± SD. Figure S4. Phytic acid in press cake and corresponding freeze‐dried sediment after protein recovery. RefBP = reference rapeseed blend pressed in a benchtop oil press. RefIP = reference rapeseed blend pressed in an industrial oil press. Data are given as the mean ± SD. Table S1. Proximate analysis on a dry basis of rapeseed press cakes from mixed blends of winter rape as references. Carbohydrates are calculated by difference and data are given as the mean ± SD. RefBP = reference rapeseed blend pressed in a benchtop oil press. RefIP = reference rapeseed blend pressed in an industrial oil press. Table S2. Extraction data and protein recovery yield from winter rape references. Data are given as the mean ± SD. RefBP = reference rapeseed blend pressed in a benchtop oil press. RefIP = reference rapeseed blend pressed in an industrial oil [file JSFA-106-4613-s001.docx]

Supplementary material


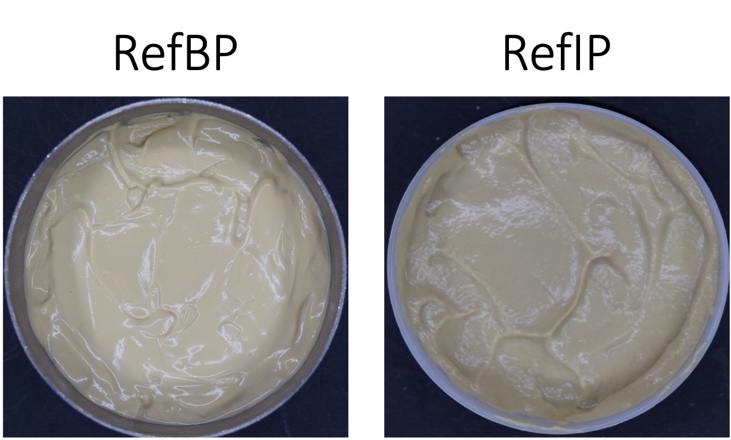


**Figure S1.** Sediments from rapeseed press cake. RefBP and RefIP are sediment extracted from press cake from a mixed blend of different rapeseed varieties where BP indicates that a benchtop oil screw press was used in the liberation of oil and IP that an industrial oil screw press was used to generate the starting material.


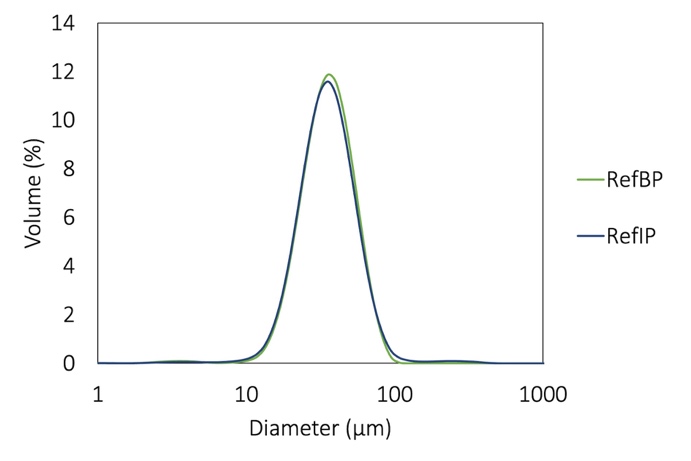

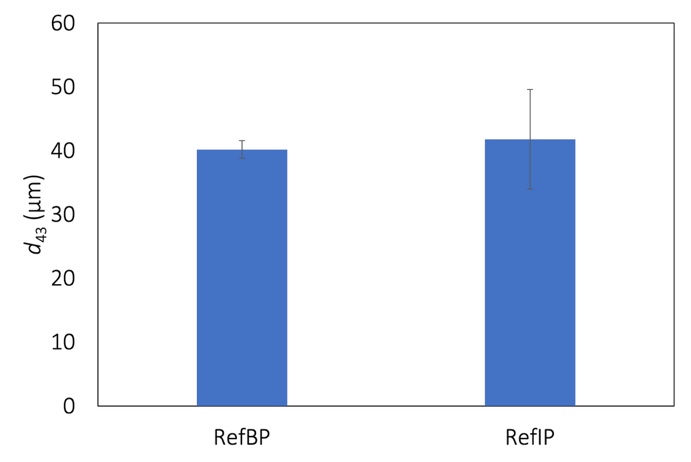


**Figure S2** Size distributions and emulsion droplet size (d_43_) in emulsions stabilized by rapeseed protein extracted from press cake from different spring varieties. Emulsions were 33% oil-in-water emulsions produced by high shear homogenization. Emulsifier concentrations in the emulsions were 8 mg protein/mL oil and data are an average from four measurements for each formulation with SD. RefBP = reference rapeseed blend pressed in a benchtop oil press. RefIP = reference rapeseed blend pressed in an industrial oil press.

**Figure S3**. Glucosinolates concentration in press cake and corresponding freeze-dried sediment after protein recovery. RefBP = reference rapeseed blend pressed in a benchtop oil press. RefIP = reference rapeseed blend pressed in an industrial oil press. Data are given as mean ± SD.


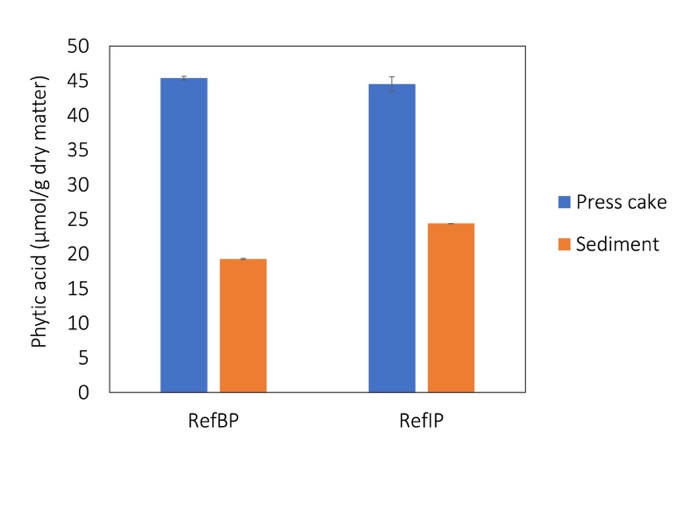


**Figure S4**. Phytic acid in press cake and corresponding freeze-dried sediment after protein recovery. RefBP = reference rapeseed blend pressed in a benchtop oil press. RefIP = reference rapeseed blend pressed in an industrial oil press. Data are given as mean ± SD.

**Table S1.** Proximate analysis on dry basis of rapeseed press cakes from mixed blends of winter rape as references. Carbohydrates are calculated by difference and data are given as mean ± SD. RefBP = reference rapeseed blend pressed in a benchtop oil press. RefIP = reference rapeseed blend pressed in an industrial oil press.

| **Rapeseed variety** | **Dry solids**  **(g/kg)** | **Protein**  **(g/kg** | **Fat**  **(g/kg** | **Carbohydrates  (g/kg)** | **Ash**  **(g/kg)** |
| --- | --- | --- | --- | --- | --- |
| *Rapeseed press cake* | | | | | |
| RefBP | 922*±*1 | 272*±*3 | 208±1 | 466±3 | 54±0 |
| RefIP | 922*±*1 | 300*±*0 | 112±2 | 528±3 | 60±0 |

**Table S2.** Extraction data and protein recovery yield from winter rape references. Data are given as mean ± SD. RefBP = reference rapeseed blend pressed in a benchtop oil press. RefIP = reference rapeseed blend pressed in an industrial oil press.

| **Rapeseed variety** | **Extraction coefficient (%)** | **Precipitation coefficient (%)** | **Protein recovery yield (%)** |
| --- | --- | --- | --- |
| RefBP | 55.2±3.5 | 55.8±9.6 | 30.4±0.2 |
| RefIP | 56.3±3.6 | 75.3*±*12 | 42.8*±*4.2 |

**Table S3.** Proximate analysis on dry basis of sediments after the protein recovery process. Carbohydrates are calculated by difference and data are given as mean ± SD. RefBP = reference rapeseed blend pressed in a benchtop oil press. RefIP = reference rapeseed blend pressed in an industrial oil press.

| **Rapeseed variety** | **Dry solids**  **(g/kg)** | **Protein**  **(g/kg)** | **Fat**  **(g/kg)** | **Carbohydrates  (g/kg)** | **Ash**  **(g/kg)** |
| --- | --- | --- | --- | --- | --- |
| RefBP | 219*±*2 | 477*±*3 | 353±11 | 146±25 | 25±1 |
| RefIP | 192±14 | 575*±*6 | 192±9 | 202±5 | 31±1 |

**Table S4.** Water holding capacity (WHC), oil holding capacity (OHC) and solubility of sediments after the protein recovery process. Data are given as mean ± SD. RefBP = reference rapeseed blend pressed in a benchtop oil press. RefIP = reference rapeseed blend pressed in an industrial oil press.

| **Rapeseed variety** | **WHC**  **(%)** | **OHC**  **(%)** | **Solubility**  **(%)** |
| --- | --- | --- | --- |
| Ref BP | 428±25 | 252±15 |  |
| RefIP | 286±5.2 | 264±4.7 |  |

**Table S5.** Concentration of glucosinolates in press cake and sediment after protein recovery from a mixed blend of winter rape. Results are expressed as mean g/kg on dry basis with SD. RefBP = reference rapeseed blend pressed in a benchtop oil press. RefIP = reference rapeseed blend pressed in an industrial oil press. Dash (-) indicates concentrations below detection limit.

| **Glucosinolate type** | **RefBP** | **RefIP** |  |  |  |  |
| --- | --- | --- | --- | --- | --- | --- |
| *Press cake* | | | | |  | |
| 4-Hydroxyglucobrassicin | 1.72±0.0 | 1.90±0.0 |  |  |  |  |
| Glucoarmoracialapicin | - | - |  |  |  |  |
| Glucobrassicin | 0.06±0.0 | 0.09±0.0 |  |  |  |  |
| Glucoiberin | - | - |  |  |  |  |
| Gluconapin | 1.06±0.0 | 1.51±0.0 |  |  |  |  |
| Gluconapoleiferin | 0.23±0.0 | 0.37±0.0 |  |  |  |  |
| Gluconasturtiin | 0.09±0.0 | 0.15±0.0 |  |  |  |  |
| Glucoraphanin | - | - |  |  |  |  |
| Neoglucobrassicin | 0.08±0.0 | - |  |  |  |  |
| Progoitrin | 1.93±0.1 | 3.21±0.1 |  |  |  |  |
| GSLs Mw 389, 448, 478 | 0.68±0.0 | 0.72±0.0 |  |  |  |  |
| Total GLSs | 5.86±0.7 | 7.96±1.0 |  |  |  |  |
| *Precipitate* | | | |  | |  |
| 4-Hydroxyglucobrassicin | - | - |  |  |  |  |
| Glucoarmoracialapicin | 0.11±0.0 | 0.12±0.0 |  |  |  |  |
| Glucobrassicin | - | - |  |  |  |  |
| Glucoiberin | - | - |  |  |  |  |
| Gluconapin | - | - |  |  |  |  |
| Gluconapoleiferin | - | - |  |  |  |  |
| Gluconasturtiin | - | - |  |  |  |  |
| Glucoraphanin | - | - |  |  |  |  |
| Neoglucobrassicin | - | - |  |  |  |  |
| Progoitrin | - | - |  |  |  |  |
| GSLs Mw 389, 448, 478 | 0.37±0.0 | 0.40±0.0 |  |  |  |  |
| Total GLSs | 0.47±0.1 | 0.51±0.1 |  |  |  |  |
